# The Role of Bioactive Compounds of *Nigella sativa* in Rheumatoid Arthritis Therapy—Current Reports

**DOI:** 10.3390/nu13103369

**Published:** 2021-09-25

**Authors:** Magdalena Zielińska, Katarzyna Dereń, Ewelina Polak-Szczybyło, Agnieszka Ewa Stępień

**Affiliations:** Department of Dietetics, Institute of Health Sciences, College for Medical Sciences, University of Rzeszow, al/Mjr. W. Kopisto 2a, 35-310 Rzeszow, Poland; mazielinska@ur.edu.pl (M.Z.); kderen@ur.edu.pl (K.D.); astepienurz@gmail.com (A.E.S.)

**Keywords:** black cumin, thymoquinone, antioxidants, autoimmune diseases

## Abstract

Black cumin (*Nigella sativa*, NS) is included in the Ranunculaceae family and is classified as a medicinal plant due to very high levels of various bioactive compounds. They determine its therapeutic effects, including anti-inflammatory, anti-allergic, anti-cancer, hypoglycemic, antioxidant, hypotensive, hypolipidemic, and immunomodulating properties. The results of scientific studies indicate a supporting role of black cumin in the treatment of autoimmune diseases, including rheumatoid arthritis, due to the health-promoting properties of its bioactive ingredients. The aim of the current article is to analyze the results of scientific publications on the role of bioactive ingredients contained in black cumin in the treatment of rheumatoid arthritis.

## 1. Introduction

Rheumatoid arthritis (RA) is a chronic, autoimmune systemic disease that reduces quality of life and shortens its duration [1,2]. RA is characterized by inflammation of the synovium of the joints, which causes pain and stiffness, and the destruction of cartilage and bone. Additional symptoms include deformity and limitations in the patient’s physical capabilities. RA affects systemic complications, such as cardiovascular, respiratory, and even psychological problems [2,3]. Symptoms of the disease usually appear between the ages of 20 and 40 and affect women more often than men, at a ratio of 2–3:1 [4]. According to a recent meta-analysis, the global incidence of RA in 1980–2018 was estimated at 0.46% [5]. Australia has the highest percentage of RA (2%) in the world [6]. In European countries, the incidence of RA is estimated from 0.35% in Serbia to 0.9% in Poland and Spain [7,8,9,10,11]. In African countries, the lowest incidence was recorded in Algeria and Egypt [12]. RA is a multifactorial disease in which various genetic, epigenetic, and environmental determinants influence the incidence of the disease in different countries [1,2,13,14]. The etiology, however, is still poorly understood and, despite the recent advances in therapy, there is still no specific, effective cure [4,15,16]. There are many identified risk factors for RA, including being female, age, smoking, and obesity [2]. Patients usually take anti-rheumatic drugs as a long-term remedy to suppress the arthritis, minimize joint damage, maintain function of the joints and remission of the disease [4]. Current clinical management of seropositive RA focuses on initiating treatment when an individual develops symptomatic and clinically recognizable arthritis, classified according to established criteria [17,18]. Several pharmaceutical therapies for the treatment of RA have been suggested, including non-steroidal anti-inflammatory drugs (NSAIDs), non-biological and biological disease-modifying anti-rheumatic drugs, immunosuppressants, and corticosteroids [19]. However, the use of these drugs is mostly associated with various side effects; therefore, recently, there has been a growing interest in complementary therapies devoid of adverse side effects [20,21]. 

Contemporary medicine has become increasingly interested in the methods of folk medicine, using medicinal plants in the prevention or supportive treatment of numerous diseases. Among others, *Nigella sativa* (NS)—also known as black cumin, black caraway, nigella, and kalonji—is highly valued in folk medicine. Black cumin seeds and oil have been used as medicinal agents in folk medicine for over 2000 years [22]. They were considered an effective drug “in every condition except death” [23]. *Nigella sativa* has anti-inflammatory, antiallergic, antitumor, hypoglycemic, antioxidant, hypotensive, hypolipidemic, immunomodulatory, nephroprotective, diuretic, anti-ulcer, and hepatoprotective effects. It also regulates acne and menstrual cycle disorders, and is used in the treatment of asthma [24,25,26,27,28,29,30,31,32,33,34,35,36,37,38,39,40,41,42,43,44,45]. *Nigella sativa* additionally shows a neuroprotective effect in Alzheimer’s and Parkinson’s diseases, depression, and epilepsy [46,47]. The results of scientific research indicate a supporting role of black cumin in the treatment of autoimmune diseases, including rheumatoid arthritis, due to the health-promoting properties of its bioactive ingredients. *Nigella sativa* seed oil is used topically in Saudi Arabia to treat joint pain and stiffness, and traditional Iranian medicine confirms its effectiveness in reducing joint pain [48]. The evaluation of its use as an alternative, natural method or adjunct to the treatment of rheumatoid arthritis has been analyzed in many scientific studies [49,50].

## 2. Bioactive Compounds in *Nigella sativa*

Black cumin belongs to the Ranunculaceae family and is one of the most important medicinal plants with a high content of bioactive compounds and numerous health properties. Its natural habitat is South Europe, North Africa, and South-West Asia. Currently, it is also cultivated in many countries around the world, including the Mediterranean region, the Middle East, and South Europe, but mainly in India, Pakistan, Syria, Turkey, and Saudi Arabia [51]. The highest quality *Nigella sativa* seeds come from Egypt due to it being the most suitable environment for their growth. The chemical composition of *Nigella sativa* seeds varies according to the cultivation method and the soil. Greenish et al. first examined the seeds of *Nigella sativa* in 1880, showing the presence of carbohydrates, proteins, fats, fiber, and vitamins [52]. Table 1 presents the results of studies on the evaluation of the content of individual nutrients in *Nigella sativa* seeds [52,53,54,55,56].

The analyses also show that the seeds of *Nigella sativa* contain mainly fatty acids—linoleic acid (64.6%) and palmitic acid (20.4%). The seed oil contains 0.4–2.5% of essential oil [57,58]. The share of soluble fiber (20.5–27.1 g/100 g) and insoluble (6.5–8.9 g/100 g) in *Nigella sativa* seeds was also determined. The total sterol content in black cumin seed oil has been shown to range from 18% to 42%, and the main sterols identified are β-sitosterol, campesterol, stigmasterol, and 5-avenasterol [55]. The total content of tocopherols in black cumin seed oil was also characterized, ranging from 9.15 to 27.92 mg/100 g, mainly α-, β-, and γ-tocopherol [55,59,60].

To date, in different varieties of black cumin seeds, many bioactive compounds have been isolated, identified, and described. These compounds are all characterized by health-promoting properties that influence their participation in supporting the treatment of patients with various diseases [61]. The most important active compounds identified are thymoquinone, thymohydroquinone, dithymoquinone, p-cymene, carvacrol, 4-terpineol, t-anethole, sesquiterpene, α-pinene, and thymol. In addition, the seeds contain three types of alkaloids (i.e., isoquinoline alkaloids), e.g., nigellicimine and nigellicimine N-oxide, pyrazole alkaloids, and rare indazole ring alkaloids, which include nigellidine and nigellicin (Table 2) [23,50,61,62,63,64,65]. Furthermore, *Nigella sativa* seeds contain saponins such as alpha-hederin, a water-soluble pentacyclic triterpene with potential anti-cancer properties. Studies have also examined the content of flavonoids; coumarins; tannins; and (in trace amounts) other compounds, including carvone, limonene and citronellol [62,63,64].

The main component of the essential oil obtained from *Nigella sativa* seeds is thymoquinone (TQ, 5-isopropyl-2-methyl-1,4-benzoquinone), which is the most bioactive compound and which exhibits a wide range of therapeutic benefits [66]. The content of individual components in the essential oil, including TQ, depends on the origin of the plant and seed storage, but also on the method of its production using supercritical CO_2_ extraction ((SC-CO_2_) −1.06; 4.07 mg/g) and the method of Soxhlet extraction (2940.43 mg/kg and 8.8 mg/g) [67]. In addition, phytochemical analyses of *Nigella sativa* seeds showed the presence of more than 100 phytonutrients; however, many of them have not yet been chemically identified and neither has their biological activity been verified.

## 3. Antioxidant, Immunomodulating, and Anti-Inflammatory Activity of Black Cumin in Rheumatoid Arthritis

*Nigella sativa* extracts and essential oils present a strong antioxidant effect [68]. Thymoquinone (TQ) is the main component of the essential oil. It supports the activity of various antioxidant enzymes, such as glutathione peroxidase, catalase, glutathione S-transferase, and glutathione reductase, acting as a neutralizer of free radicals and peroxides (reactive oxygen species) [68,69,70]. These compounds also inhibit cyclooxygenase and 5-lipoxygenase in the arachidonic acid pathway cascade. As a result of the thin-layer chromatography technique, thymoquinone, carvacrol, t-anethole, and 4-terpineol were isolated from black cumin seeds, and their valuable antioxidant activity of synergistic nature was determined using the DPPH (2,2-diphenyl-1-picrylhydrazyl) test [71]. The studies confirmed that the TQ contained in *Nigella sativa* oil administered to RA patients shows a protective effect against rheumatoid arthritis and reduces the score on the activity scale of arthritis (disease activity score (DAS) 28) and bone resorption. Hadi et al. showed that the components of black cumin simultaneously reduce oxidative stress and influence the inflammatory process by inhibiting the activity of NF-κB, which is induced by TNF-α, IL-6, and other cytokines, leading to the persistence of inflammation [72]. The increased level and activity of pro-inflammatory cytokines, namely TNF-α, IL-1, and IL-6, lead to uncontrolled inflammation that damages bone and cartilage and causes symptoms of RA [73]. Furthermore, *Nigella sativa* exhibits immunomodulatory activity, which is another factor that possibly influences RA [74]. It has been shown that *Nigella sativa* can improve the immune response (especially T lymphocytes) and increase the ratio of T helper lymphocytes to suppressor T lymphocytes, increasing, in turn, the activity of NK cells [75,76]. In addition to the inhibitory effect of TQ on eicosanoid production, it has been suggested that TQ retains its anti-inflammatory effect by inhibiting various pro-inflammatory transcription factors, such as NF-κB/STAT3, by inducing several stimuli, including cytokines and free radicals. According to the researchers, the results of studies on animals with arthritis in 2005 indicate that NF-κB played a dominant role in the development of arthritis [77]. Moreover, activation of NF-κB has been observed in the synovial tissue of RA patients in both early and late stages of this disease [73]. Therefore, inhibitors of NF-κB are considered to be therapeutic and suitable for the treatment of RA. The use of TQ can reduce pro-inflammatory responses mainly by modulating the activity of NF-κB and inhibiting the production of IL-1β, IL-6, TNF-α, and IFN-γ [78]. Furthermore, the anti-inflammatory properties of *Nigella sativa* in RA are supported by studies showing a suppressive effect of TQ on nitric oxide (NO) production [79]. NO has pro-inflammatory activity, and it is produced from activated macrophages in the case of an inflammatory reaction. Inflammatory cytokines in chondrocytes can increase the activity of inducible nitric oxide synthesis (iNOS) and result in the production of NO. It has been declared that the activity of iNOS and plasma NO levels are higher in RA patients in comparison to healthy controls [80]. Thus, agents that prevent additional NO production may have a therapeutic effect on arthritis by inhibiting the destruction of cartilage [81].

## 4. *Nigella sativa* and Rheumatoid Arthritis In Vitro, Animal, and Clinical Studies

The results of in vitro studies indicate the influence of *Nigella sativa* on the course of RA by demonstrating the strong anti-inflammatory and antioxidant properties of TQ [82]. These results were found in a 2015 study investigating the effects of *Nigella sativa* on RA. Thy indicate that treatment of human synovial fibroblasts in RA with 1–5 µM thymoquinone can cause apoptosis by blocking the expression of myeloid leukemia (Mcl)–1 and inhibiting the TNF-α-induced production of IL-6 and IL-8. TQ therapy can further reduce TNF-α-induced intercellular adhesion molecule (ICAM)–1 and vascular cell adhesion molecule (VCAM)–1 expression, reduce cadherin (Cad)–11 expression, and inhibit TNF-α-induced phosphorylation of p38 and c–Jun N-terminal kinase (JNK) in a dose-dependent manner. TQ administration is also illustrated to prevent phosphorylation and the subsequent activation of TNF-α-induced apoptosis-mediated signal kinase (ASK)–1 to slow down TNF-α signaling and inhibit p38/JNK-mediated expression [83]. Thymoquinone animal studies have also confirmed the beneficial effects of *Nigella sativa* on RA (Table 3) [84,85,86,87,88,89].

Taking into account other complications of RA, such as respiratory or psychological complications, it is worth noting that black cumin seed also has a protective effect [24,45,56,93,94,95,96]. The use of *Nigella sativa* in this group of patients is also beneficial due to its properties, which reduce the risk of cardiovascular diseases. They result from a comprehensive antioxidant effect, blocking the calcium channel, lowering blood pressure as well as levels of lactate dehydrogenase (LDH) and plasma creatine kinase (CK), and reducing oxidative damage. TQ has been found to improve the lipid profile and protect against the development of atherosclerosis, as well as normalizing the renin–angiotensin–aldosterone (RAA) system and improving endothelial function in the arterial wall [97,98,99].

An important issue regarding the use of the oil in RA *Nigella sativa* therapy is its safety for patients. Assessment of the side effects of the oral use of *Nigella sativa* oil at the recommended doses did not reveal any adverse effects [72,91,92,100]. Hadi and Kheirouri et al. determined the optimal dose of *Nigella sativa* in rheumatoid arthritis at 500 mg, twice a day for 8 weeks [72,91]. In addition to thymoquinone, there are many active compounds that synergistically affect the health-promoting properties of this plant. It is important to standardize the bioactive compounds present in *Nigella sativa* supplements to ensure quality, effectiveness, and safety when taking these preparations for patients [101]. There are still no standards defining the minimum acceptable content of active substances in products from *Nigella sativa* [102]. Currently, scientific research indicates analytical techniques that can potentially be used to assess the quality of seeds and the amount of active ingredients contained in them. The standardization of the raw material affects the medicinal preparations obtained from them. These methods are accepted by the WHO, FDA, and China Food and Drug Administration [103]. Yun et al. mentioned ultra-performance liquid chromatography coupled with electrospray time-of-flight mass spectrometry (UPLC-Q-TOF/MS) and the HPLC fingerprinting method as the most optimal methods for detecting bioactive compounds present in black cumin seeds, even those unstable and of low molecular weight. They propose the use of these methods for the standardization and control of the quality of *Nigella Sativa* seeds, extract, and oil [103]. Ahmad et al., for the analysis of finished black cumin preparations, recommend a stable method of thin-layer chromatography (HPTLC) [102].

## 5. Conclusions

*Nigella sativa* has analgesic, anti-inflammatory, immunomodulatory, and RA lesion-reducing effects in in vitro, animal, and human clinical trials. Clinical studies have confirmed the efficacy and safety of *Nigella sativa* oil in the treatment of pain and inflammation in patients suffering from rheumatoid arthritis. *Nigella sativa* has also been shown to reduce oxidative stress in RA patients, and supplementation with the seed extract may be a beneficial adjunctive therapy in this patient population. Thymoquinone, the active ingredient present in *Nigella sativa*, has a beneficial effect on clinical, inflammatory, oxidative, and immune parameters in RA. The results of the study justify the need for further clinical trials.

## Figures and Tables

**Table 1:** Nutritional composition of *Nigella sativa* seeds.

**Nutritional Composition**	**Contents [%]**
Water	3.8–7.0
Proteins (phenylalanine, leucine, glutamic acid, glycine, lysine, arginine, valine, aspartic acid, histidine isoleucine, methionine, and threonine)	18.59–31.2
Fats (linoleic acid, oleic acid, eicodiamic acid, myristoleic acid, myristic acid, stearic acid, palmitic acid, sterols (lanosterol, campesterol, β-sitosterol, avenasterol, and stigmasterol), and arachidic acid)	22.0–56.4
Carbohydrates (xylose, arabinose, rhamnose, and glucose)	24.9–40.0
Dietary fiber	3.7–4.7
**Fat-Soluble Vitamins**	**[mg/kg]**
DL-α-tocopherol	0.177
DL-β-tocopherol	9.027
DL-γ-tocopherol	5.427
All trans-retinol	0.277
**Water-Soluble Vitamins**	**[mg/kg]**
Vitamin B1	13–18
Vitamin B6	4–15
Niacin	33–97
Folic acid	400–870
**Minerals**	**[mg/100 g]**
Iron	9.10–15.40
Copper	1.50–3.75
Sodium	41.20–55.0
Potassium	442.3–675.0
Calcium	154.4–305.0
Zinc	3.36–6.60
Phosphor	378.12–576.90
Magnesium	134.90–147.05

**Table 2:** The content of the most important bioactive compounds in the seeds of *Nigella sativa*.

Active Compounds	Contents [%]
Thymoquinone	30–48%
Thymohydroquinone, dithymoquinone, and p-cymene	7–15%
Carvacrol	6–12%
4-Terpineol	2–7%
T-anethol	1–4%
Longifolene (a sesquiterpene)	1–8%
Nigellicimine, N-tlenek nigellicimine, nigellidine, nigellicine, α-hederin, saponin, carvone, limonene, and citronellol	<1% (trace amounts)

**Table 3:** Characteristics of animal studies of the effects of *Nigella sativa* on rheumatoid arthritis.

Intervention	Dose/Duration	Study Group	Results	Bibliography
Thymoquinone	10 mg/kg body weight /20 days	40 male rats Sprague–Dawley	1. TQ treatment reduced macroscopic arthritis score, CRP levels, synovitis, pannus formation, and bone erosion. 2. The level of TLR2, TLR4, IL-1, NF-κB mRNA, and TNF-α was also decreased. 3. TQ also normalized hematology markers and showed no signs of hepatotoxicity or nephrotoxicity.	Arjumand et al. (2019) [84]
*Nigella sativa* oil	1.82 mL/kg or 0.91 mL/kg (this corresponds to 1596 and 798 mg/kg respectively) /25 days	Rats with arthritis by using Freund’s complete adjuvant (CFA)	1. Significant reduction in paw volume compared to the control group. 2. Significant antinociceptive effect in the contralateral hind paw compared to the control group. 3. No significant antinociceptive activity in the inoculated hind paw compared to the CFA control group.	Nasuti et al. (2019) [85]
Thymoquinone	2 mg/kg body weight /15 days	Ratswith arthritis	Significant reductions in paw weight and histopathology score (e.g., inflammatory cells and synovial hyperplasia) compared to the arthritic control.	Faisal et al. (2018) [86]
Thymoquinone	2 mg/kg body weight /15 days	32 female Sprague–Dawley rats	Significant reduction in TLC (total leukocyte count) and clinical assessment of inflammation, and improvement in blood urea and serum creatinine compared to arthritis control.	Faisal et al. (2015) [87]
Thymoquinone	2 mg/kg body weight /15 days	Rats with arthritis	Significant reduction in TLC (total leukocyte count) and inflammatory cell counts compared to the arthritic control group.	Faisal et al. (2015) [88]
Thymoquinone	2 mg/kg body weight /15 days	Rats with arthritis	Significant decrease in the clinical assessment of inflammation and TLC (total leukocyte count) and normalization of DLC (differential leukocyte count).	Faisal et al. (2015) [89]

Pain and inflammation are the first clinical signs of rheumatoid arthritis to be considered for treatment, and this aspect has been analyzed. The results of clinical trials in humans on the effectiveness of *Nigella sativa* oil in the treatment of pain and inflammation in RA are presented in Table 4 [72,90,91,92].

**Table 4:** Characteristics of human studies of the effects of *Nigella sativa* on rheumatoid arthritis.

Intervention	Dose/Duration	Study Group	Results	Bibliography
Black cumin oil capsules	1 g/day (2 capsules, 500 mg/day) /8 weeks	*n* = 50 patients (39 complete completion) (intervention group *n* = 23; placebo group *n* = 16)	1. Significant decrease in the DAS-28 score compared to the placebo group. 2. Serum IL-10 level was increased in the intervention group (*p* < 0.01), and there was a reduction in MDA and NO in serum in comparisonto the baseline value (*p* < 0.05). 3. No significant differencesin serum IL-10, TNF-α, MDA, SOD, catalase, TAC, and NOcompared to the placebo group.	Hadi et al.(2016) [72]
Black cumin oil capsules	1 g/day (2 capsules, 500 mg/day) /8 weeks	*n* = 43 women (intervention group *n* = 23; placebo group *n* = 20)	1. Significant reduction in DAS-28 and CD8 + score comparedto the placebo group. 2. Significant increase in CD4 + / CD8 + ratio and percentage of CD4 + CD25 + regulatory T cells compared tothe placebo group. 3. No significant changes in the percentage of CD4 + T cells compared to the placebo group.	Kheirouri et al. (2016) [91]
1st group: low-calorie diet with 3 g/day of *Nigella sativa* oil. 2nd group: low calorie diet with 3 g/day placebo for 8 weeks.	3 g/day /8 weeks	n = 90 volunteers (84 years of age completed the study women) (intervention group *n* = 43; placebo group *n* = 41)	1. *Nigella sativa* oil lowered levels of tumor necrosis factor TNF-α and C-reactive protein with high sensitivity compared to the placebo group. 2. There were no significant changes in the levels of interleukin-6 in the *Nigella sativa* group compared to the placebo group.	Mahdavi et al. (2016) [92]

## References

[1] Smolen J.S., Aletaha D., Barton A., Burmester G.R., Emery P., Firestein G.S., Kavanaugh A., McInnes I.B., Solomon D.H., Strand V. (2018). Rheumatoid arthritis. Nat. Rev. Dis. Primers.

[2] Giannini D., Antonucci M., Petrelli F., Bilia S., Alunno A., Puxeddu I. (2020). One year in review 2020: Pathogenesis of rheumatoid arthritis. Clin. Exp. Rheumatol..

[3] Vallerand I.A., Patten S.B., Barnabe C. (2019). Depression and the risk of rheumatoid arthritis. Curr. Opin. Rheumatol..

[4] Alam J., Jantan I., Bukhari S.N.A. (2017). Rheumatoid arthritis: Recent advances on its etiology; role of cytokines and pharmacotherapy. Biomed. Pharmacother..

[5] Almutairi K., Nossent J., Preen D., Keen H., Inderjeeth C. (2021). The global prevalence of rheumatoid arthritis: A meta-analysis based on a systematic review. Rheumatol. Int..

[6] Australian Institute of Health and Welfare Rheumatoid Arthritis. https://www.aihw.gov.au/reports/arthritis-other-musculoskeletal-conditions/rheumatoid-arthritis/contents/who-gets-rheumatoid-arthritis.

[7] Seoane-Mato D., Sánchez-Piedra C., Silva-Fernández L., Sivera F., Blanco F.J., Pérez Ruiz F., Juan-Mas A., Pego-Reigosa J.M., Martí N.Q., Cortés Verdú R. (2019). Prevalence of rheumatic diseases in adult population in Spain (EPISER 2016 study): Aims and methodology. Reumatol. Clin..

[8] Batko B., Stajszczyk M., Świerkot J., Urbański K., Raciborski F., Jędrzejewski M., Wiland P. (2019). Prevalence and clinical characteristics of rheumatoid arthritis in Poland: A nationwide study. Arch. Med. Sci..

[9] Roux C.H., Saraux A., Le Bihan E., Fardellone P., Guggenbuhl P., Fautrel B., Masson C., Chary-Valckenaere I., Cantagrel A., Juvin R. (2007). Rheumatoid arthritis and spondyloarthropathies: Geographical variations in prevalence in France. J. Rheumatol..

[10] Rossini M., Rossi E., Bernardi D., Viapiana O., Gatti D., Idolazzi L., Caimmi C., Derosa M., Adami S. (2014). Prevalence and incidence of rheumatoid arthritis in Italy. Rheumatol. Int..

[11] Zlatković-Švenda M.I., Stojanović R.M., Šipetić-Grujičić S., Guillemin F. (2014). Prevalence of rheumatoid arthritis in Serbia. Rheumatol. Int..

[12] Slimani S., Ladjouze-Rezig A. (2014). Prevalence of rheumatoid arthritis in an urban population of Algeria: A prospective study. Rheumatology.

[13] Liao K.P., Alfredsson L., Karlson E.W. (2009). Environmental influences on risk for rheumatoid arthritis. Curr. Opin. Rheumatol..

[14] Tobón G.J., Youinou P., Saraux A. (2010). The environment; geo-epidemiology; and autoimmune disease: Rheumatoid arthritis. J. Autoimmun..

[15] Guo Q., Wang Y., Xu D., Nossent J., Pavlos N.J., Xu J. (2018). Rheumatoid arthritis: Pathological mechanisms and modern pharmacologic therapies. Bone Res..

[16] Pabón-Porras M.A., Molina-Ríos S., Flórez-Suárez J.B., Coral-Alvarado P.X., Méndez-Patarroyo P., Quintana-López G. (2019). Rheumatoid arthritis and systemic lupus erythematosus: Pathophysiological mechanisms related to innate immune system. SAGE Open Med..

[17] Singh J.A., Saag K.G., Bridges S.L., Akl E.A., Bannuru R.R., Sullivan M.C., Vaysbrot E., McNaughton C., Osani M., Shmerling R.H. (2016). 2015 American College of Rheumatology Guideline for the Treatment of Rheumatoid Arthritis. Arthritis Rheumatol..

[18] Aletaha D., Neogi T., Silman A.J., Funovits J., Felson D.T., Bingham C.O., Birnbaum N.S., Burmester G.R., Bykerk V.P., Cohen M.D. (2010). 2010 Rheumatoid arthritis classification criteria: An American College of Rheumatology/European League Against Rheumatism collaborative initiative. Arthritis Rheum..

[19] Sparks J.A. (2019). Rheumatoid Arthritis. Ann. Intern. Med..

[20] Lindler B.N., Long K.E., Taylor N.A., Lei W. (2020). Use of Herbal Medications for Treatment of Osteoarthritis and Rheumatoid Arthritis. Medicines.

[21] Adib-Hajbaghery M., Rafiee S. (2018). Medicinal plants use by elderly people in Kashan; Iran. Nurs. Midwifery Stud..

[22] Darakhshan S., Bidmeshki Pour A., Hosseinzadeh Colagar A., Sisakhtnezhad S. (2015). Thymoquinone and its therapeutic potentials. Pharmacol. Res..

[23] Ahmad A., Husain A., Mujeeb M., Khan S.A., Najmi A.K., Siddique N.A., Damanhouri Z.A., Anwar F. (2013). A review on therapeutic potential of Nigella sativa: A miracle herb. Asian Pac. J. Trop. Biomed..

[24] Amin B., Hosseinzadeh H. (2016). Black Cumin (Nigella sativa) and Its Active Constituent; Thymoquinone: An Overview on the Analgesic and Anti-inflammatory Effects. Planta Med..

[25] Shaterzadeh-Yazdi H., Noorbakhsh M.F., Hayati F., Samarghandian S., Farkhondeh T. (2018). Immunomodulatory and Anti-inflammatory Effects of Thymoquinone. Cardiovasc. Hematol. Disord. Drug Targets.

[26] Gholamnezhad Z., Shakeri F., Saadat S., Ghorani V., Boskabady M.H. (2019). Clinical and experimental effects of Nigella sativa and its constituents on respiratory and allergic disorders. Avicenna J. Phytomed..

[27] Saadat S., Aslani M.R., Ghorani V., Keyhanmanesh R., Boskabady M.H. (2021). The effects of Nigella sativa on respiratory; allergic and immunologic disorders; evidence from experimental and clinical studies; a comprehensive and updated review. Phytother. Res..

[28] Imran M., Rauf A., Khan I.A., Shahbaz M., Qaisrani T.B., Fatmawati S., Abu-Izneid T., Imran A., Rahman K.U., Gondal T.A. (2018). Thymoquinone: A novel strategy to combat cancer: A review. Biomed. Pharmacother..

[29] Korak T., Ergül E., Sazci A. (2020). Nigella sativa and Cancer: A Review Focusing on Breast Cancer; Inhibition of Metastasis and Enhancement of Natural Killer Cell Cytotoxicity. Curr. Pharm. Biotechnol..

[30] Askari G., Rouhani M.H., Ghaedi E., Ghavami A., Nouri M., Mohammadi H. (2019). Effect of Nigella sativa (black seed) supplementation on glycemic control: A systematic review and meta-analysis of clinical trials. Phytother. Res..

[31] Mahmoodi M.R., Mohammadizadeh M. (2020). Therapeutic potentials of Nigella sativa preparations and its constituents in the management of diabetes and its complications in experimental animals and patients with diabetes mellitus: A systematic review. Complement. Ther. Med..

[32] Ardiana M., Pikir B.S., Santoso A., Hermawan H.O., Al-Farabi M.J. (2020). Effect of Nigella sativa Supplementation on Oxidative Stress and Antioxidant Parameters: A Meta-Analysis of Randomized Controlled Trials. Sci. World J..

[33] Fallah Huseini H., Amini M., Mohtashami R., Ghamarchehre M.E., Sadeqhi Z., Kianbakht S., Fallah Huseini A. (2013). Blood pressure lowering effect of Nigella sativa L. seed oil in healthy volunteers: A randomized; double-blind; placebo-controlled clinical trial. Phytother. Res..

[34] Pakkir Maideen N.M., Balasubramanian R., Ramanathan S. (2020). Nigella Sativa (Black seeds); a Potential herb for the Pharmacotherapeutic Management of Hypertension-A Review. Curr. Cardiol. Rev..

[35] Asgary S., Sahebkar A., Goli-Malekabadi N. (2015). Ameliorative effects of Nigella sativa on dyslipidemia. J. Endocrinol. Investig..

[36] Majdalawieh A.F., Fayyad M.W. (2015). Immunomodulatory and anti-inflammatory action of Nigella sativa and thymoquinone: A comprehensive review. Int. Immunopharmacol..

[37] Benhelima A., Kaid-Omar Z., Hemida H., Benmahdi T., Addou A. (2016). Nephroprotective And Diuretic Effect of Nigella Sativa L Seeds Oil On Lithiasic Wistar Rats. Afr. J. Tradit. Complement. Altern. Med..

[38] Dajani E.Z., Shahwan T.G., Dajani N.E. (2016). Overview of the preclinical pharmacological properties of Nigella sativa (black seeds): A complementary drug with historical and clinical significance. J. Physiol. Pharmacol..

[39] Tekbas A., Huebner J., Settmacher U., Dahmen U. (2018). Plants and Surgery: The Protective Effects of Thymoquinone on Hepatic Injury-A Systematic Review of In Vivo Studies. Int. J. Mol. Sci..

[40] Noorbakhsh M.F., Hayati F., Samarghandian S., Shaterzadeh-Yazdi H., Farkhondeh T. (2018). An Overview of Hepatoprotective Effects of Thymoquinone. Recent Pat. Food Nutr. Agric..

[41] Samadipour E., Rakhshani M.H., Kooshki A., Amin B. (2020). Local Usage of Nigella sativa Oil as an Innovative Method to Attenuate Primary Dysmenorrhea: A Randomized Double-blind Clinical Trial. Oman Med. J..

[42] Soleymani S., Zargaran A., Farzaei M.H., Iranpanah A., Heydarpour F., Najafi F., Rahimi R. (2020). The effect of a hydrogel made by Nigella sativa L. on acne vulgaris: A randomized double-blind clinical trial. Phytother. Res..

[43] Koshak A., Wei L., Koshak E., Wali S., Alamoudi O., Demerdash A., Qutub M., Pushparaj P.N., Heinrich M. (2017). Nigella sativa Supplementation Improves Asthma Control and Biomarkers: A Randomized; Double-Blind; Placebo-Controlled Trial. Phytother. Res..

[44] Zhang K. (2020). Is Nigella sativa supplementation effective for asthma?. Am. J. Emerg. Med..

[45] He T., Xu X. (2020). The influence of Nigella sativa for asthma control: A meta-analysis. Am. J. Emerg. Med..

[46] Samarghandian S., Farkhondeh T., Samini F. (2018). A Review on Possible Therapeutic Effect of Nigella sativa and Thymoquinone in Neurodegenerative Diseases. CNS Neurol. Disord. Drug Targets.

[47] Cobourne-Duval M.K., Taka E., Mendonca P., Soliman K.F.A. (2018). Thymoquinone increases the expression of neuroprotective proteins while decreasing the expression of pro-inflammatory cytokines and the gene expression NFκB pathway signaling targets in LPS/IFNγ -activated BV-2 microglia cells. J. Neuroimmunol..

[48] Houghton P.J., Zarka R., de las Heras B., Hoult J.R. (1995). Fixed oil of Nigella sativa and derived thymoquinone inhibit eicosanoid generation in leukocytes and membrane lipid peroxidation. Planta Med..

[49] Chen Q.L., Chen X.Y., Zhu L., Chen H.B., Ho H.M., Yeung W.-P., Zhao Z.-Z., Yi T. (2016). Review on Saussurea laniceps; a potent medicinal plant known as “snow lotus”: Botany; phytochemistry and bioactivities. Phytochem. Rev..

[50] Yi T., Zhu L., Zhu G.Y., Tang Y.N., Xu J., Fan J.-Y., Zhao Z.-Z., Chen H.-B. (2016). HSCCC-based strategy for preparative separation of in vivo metabolites after administration of an herbal medicine: Saussurea laniceps; a case study. Sci. Rep..

[51] Kooti W., Hasanzadeh-Noohi Z., Sharafi-Ahvazi N., Asadi-Samani M., Ashtary-Larky D. (2016). Phytochemistry; pharmacology; and therapeutic uses of black seed (Nigella sativa). Chin. J. Nat. Med..

[52] Greenish H.G. (1880). Contribution to the chemistry of Nigella sativa. Pharmac. J. Trans..

[53] Al-Jassir M.S. (1992). Chemical composition and microflora of black cumin (Nigella sativa L.) seeds growing in Saudi Arabia. Food Chem..

[54] Al-Saleh I.A., Billedo G., El–Doush I.I. (2006). Levels of selenium; DLa-tocopherol; DL-g-tocopherol; all-trans-retinol; thymoquinone and thymol in different brands of Nigella sativa seeds. J. Food Comp. Anal..

[55] Cheikh-Rouhou S., Besbes S., Hentati B., Blecker C., Deroanne C., Attia H. (2007). Nigella sativa L.: Chemical composition and physicochemical characteristics of lipid fraction. Food Chem..

[56] Ramadan M.F., Morsel J.T. (2003). Analysis of glycolipids from black cumin (Nigella sativa L.); coriander (Coriandrum sativum L.) and niger (Guizotia abyssinica Cass.) oilseeds. Food Chem..

[57] Mamun M.A., Absar N. (2018). Major nutritional compositions of black cumin seeds cultivated in Bangladesh and the physicochemical characteristics of its oil. Int. Food Res. J..

[58] Ghahramanloo K.H., Kamalidehghan B., Akbari Javar H., Teguh Widodo R., Majidzadeh K., Noordin M.I. (2017). Comparative analysis of essential oil composition of iranian and indian nigella sativa L. Extracted using supercritical fluid extraction and solvent extraction. Drug Design. Dev. Ther..

[59] Pop R.M., Trifa A.P., Popolo A., Chedea V.S., Militaru C., Bocsan I.C., Buzoianu A.D. (2020). Nigella sativa: Valuable perspective in the management of chronic diseases. Iran J. Basic Med. Sci..

[60] Juhaimi F., Matthäus B., Ghafoor K., ElBabiker E.F., Ozcan F.F. (2016). Fatty acids; tocopherols; minerals contents of Nigella sativa and Trigonella foenum-graecum seed and seed oils. Rivista Italiana Delle Sostanze Grasse.

[61] Shafiq H., Ahmad A., Masud T., Kaleem M. (2014). Cardio-protective and anti-cancer therapeutic potential of Nigella sativa. Iran J. Basic Med. Sci..

[62] Adamska A., Stefanowicz-Hajduk J., Ochocka J.R. (2019). Alpha-Hederin; the Active Saponin of Nigella sativa; as an Anticancer Agent Inducing Apoptosis in the SKOV-3 Cell Line. Molecules.

[63] Isik S., Kartal M., Erdem S.A. (2017). Quantitative analysis of thymoquinone in Nigella Sativa, L. (Black Cumin) seeds and commercial seed oils and seed oil capsules from Turkey. Ankara Üniversitesi Eczacılık Fakültesi Dergisi.

[64] Tavakkoli A., Mahdian V., Razavi B.M., Hosseinzadeh H. (2017). Review on Clinical Trials of Black Seed (Nigella sativa) and Its Active Constituent; Thymoquinone. J. Pharmacopunct..

[65] Shomar B. (2012). Major and trace elements in Nigella sativa provide a potential mechanism for its healing effects. J. Med. Plants Res..

[66] Haseena S., Aithal M., Das K.K., Saheb S.H. (2015). Phytochemical analysis of Nigella sativa and its effect on reproductive system. J. Pharm. Sci. Res..

[67] Herlina Aziz S.A., Kurniawati A., Faridah D.N. (2017). Changes of thymoquinone; thymol; and malondialdehyde content of black cumin (Nigella sativa L.) in response to Indonesia tropical. J. Biosci..

[68] Ahmad M.F., Ahmad F.A., Ashraf S.A., Saad H.H., Wahab S., Khan M.I., Ali M., Mohan S., Hakeem K.R., Athar M.T. (2021). An updated knowledge of Black seed (Nigella sativa Linn.): Review of phytochemical constituents and pharmacological properties. J. Herb. Med..

[69] Mansour M.A., Nagi M.N., El-Khatib A.S., Al-Bekairi A.M. (2002). Effects of thymoquinone on antioxidant enzyme activities; lipid peroxidation and DT-diaphorase in different tissues of mice: A possible mechanism of action. Cell Biochem. Funct..

[70] Khalife K.H., Lupidi G. (2007). Nonenzymatic reduction of thymoquinone in physiological conditions. Free Radic. Res..

[71] Burits M., Bucar F. (2000). Antioxidant activity of Nigella sativaessential oil. Phytother. Res..

[72] Hadi V., Kheirouri S., Alizadeh M., Khabbazi A., Hosseini H. (2016). Effects of Nigella sativa oil extract on inflammatory cytokine response and oxidative stress status in patients with rheumatoid arthritis: A randomized; double-blind; placebo-controlled clinical trial. Avicenna J. Phytomed..

[73] Aravilli R.K., Vikram S.L., Kohila V. (2017). Phytochemicals as potential antidotes for targeting NF-κB in rheumatoid arthritis. 3 Biotech.

[74] Akram Khan M., Afzal M. (2016). Chemical composition of Nigella sativa Linn: Part 2 Recent advances. Inflammopharmacology.

[75] Swamy S.M., Tan B.K. (2000). Cytotoxic and immunopotentiating effects of ethanolic extract of Nigella sativa L. seeds. J. Ethnopharmacol..

[76] Abdel-Zaher A.O., Abdel-Rahman M.S., Elwasei F.M. (2011). Protective effect of Nigella sativa oil against tramadol-induced tolerance and dependence in mice: Role of nitric oxide and oxidative stress. Neurotoxicology.

[77] Mor A., Abramson S.B., Pillinger M.H. (2005). The fibroblast-like synovial cell in rheumatoid arthritis: A key player in inflammation and joint destruction. Clin. Immunol..

[78] Bordoni L., Fedeli D., Nasuti C., Maggi F., Papa F., Wabitsch M., De Caterina R., Gabbianelli R. (2019). Antioxidant and Anti-Inflammatory Properties of Nigella sativa Oil in Human Pre-Adipocytes. Antioxidants.

[79] Umar S., Zargan J., Umar K., Ahmad S., Katiyar C.K., Khan H.A. (2012). Modulation of the oxidative stress and inflammatory cytokine response by thymoquinone in the collagen induced arthritis in Wistar rats. Chem. Biol. Interact..

[80] Romas E., Sims N.A., Hards D.K., Lindsay M., Quinn J.W., Ryan P.F., Dunstan C.R., Martin T.J., Gillespie M.T. (2002). Osteoprotegerin reduces osteoclast numbers and prevents bone erosion in collagen-induced arthritis. Am. J. Pathol..

[81] Shukla M., Gupta K., Rasheed Z., Khan K.A., Haqqi T.M. (2008). Bioavailable constituents/metabolites of pomegranate (Punica granatum L) preferentially inhibit COX2 activity ex vivo and IL-1beta-induced PGE2 production in human chondrocytes in vitro. J. Inflamm..

[82] Vaillancourt F., Silva P., Shi Q., Fahmi H., Fernandes J.C., Benderdour M. (2011). Elucidation of molecular mechanisms underlying the protective effects of thymoquinone against rheumatoid arthritis. J. Cell Biochem..

[83] Umar S., Hedaya O., Singh A.K., Ahmed S. (2015). Thymoquinone inhibits TNF-α-induced inflammation and cell adhesion in rheumatoid arthritis synovial fibroblasts by ASK1 regulation. Toxicol. Appl. Pharmacol..

[84] Arjumand S., Shahzad M., Shabbir A., Yousaf M.Z. (2019). Thymoquinone attenuates rheumatoid arthritis by downregulating TLR2; TLR4; TNF-α; IL-1; and NFκB expression levels. Biomed. Pharmacother..

[85] Nasuti C., Fedeli D., Bordoni L., Piangerelli M., Servili M., Selvaggini R., Gabbianelli R. (2019). Anti-inflammatory; anti-arthritic and anti-nociceptive activities of Nigella sativa oil in a rat model of arthritis. Antioxidants.

[86] Faisal R., Ahmad N., Fahad Y.S., Chiragh S. (2018). Anti-Arthritic Effect Of Thymoquinone In Comparison With Methotrexate On Pristane Induced Arthritis In Female Sprague Dawley Rats. J. Ayub Med. Coll. Abbottabad.

[87] Faisal R., Shinwari L., Jehangir T. (2015). Comparison of the Therapeutic Effects of Thymoquinone and Methotrexate on Renal Injury in Pristane Induced Arthritis in Rats. J. Coll. Physicians Surg. Pak..

[88] Faisal R., Imran U. (2015). Comparative evaluation of thymoquinone and methotrexate in lung inflammation in murine model of rheumatiod arthritis. J. Postgrad. Med. Inst..

[89] Faisal R., Chiragh S., Popalzai A.J., Rehman K.U. (2015). Anti-inflammatory effect of thymoquinone in comparison with methotrexate on pristane induced arthritis in rats. J. Pak. Med. Assoc..

[90] Mahboubi M., Mohammad Taghizadeh Kashani L., Mahboubi M. (2018). Nigella sativa fixed oil as alternative treatment in management of pain in arthritis rheumatoid. Phytomedicine.

[91] Kheirouri S., Hadi V., Alizadeh M. (2016). Immunomodulatory Effect of Nigella sativa Oil on T Lymphocytes in Patients with Rheumatoid Arthritis. Immunol. Investig..

[92] Mahdavi R., Namazi N., Alizadeh M., Farajnia S. (2016). Nigella sativa oil with a calorie-restricted diet can improve biomarkers of systemic inflammation in obese women: A randomized double-blind; placebo-controlled clinical trial. J. Clin. Lipidol..

[93] Al Disi S.S., Anwar M.A., Eid A.H. (2016). Anti-hypertensive Herbs and their Mechanisms of Action: Part, I. Front. Pharmacol..

[94] Hannan M.A., Rahman M.A., Sohag A.A.M., Uddin M.J., Dash R., Sikder M.H., Rahman S., Timalsina B., Munni Y.A., Sarker P.P. (2021). Black Cumin (Nigella sativa L.): A Comprehensive Review on Phytochemistry; Health Benefits; Molecular Pharmacology; and Safety. Nutrients.

[95] Kohandel Z., Farkhondeh T., Aschner M., Samarghandian S. (2021). Anti-inflammatory effects of thymoquinone and its protective effects against several diseases. Biomed. Pharmacother..

[96] Khazdair M.R., Anaeigoudari A., Hashemzehi M., Mohebbati R. (2018). Neuroprotective potency of some spice herbs; a literature review. J. Tradit. Complement. Med..

[97] Verma T., Sinha M., Bansal N., Yadav S.R., Shah K., Chauhan N.S. (2021). Plants Used as Antihypertensive. Nat. Prod. Bioprospect..

[98] Jaarin K., Foong W.D., Yeoh M.H., Kamarul Z.Y., Qodriyah H.M., Azman A., Zuhair J.S., Juliana A.H., Kamisah Y. (2015). Mechanisms of the antihypertensive effects of Nigella sativa oil in L-NAME-induced hypertensive rats. Clinics.

[99] Musharraf H.M., Arman M.S.I. (2018). Prophetic medicine is the cheapest; safest and the best remedy in the prevention and treatment of hypertension (high blood pressure)—A mini review. Int. J. Mol. Biol..

[100] Kooshki A., Forouzan R., Rakhshani M.H., Mohammadi M. (2016). Effect of Topical Application of Nigella Sativa Oil and Oral Acetaminophen on Pain in Elderly with Knee Osteoarthritis: A Crossover Clinical Trial. Electron. Physician.

[101] Sachan A., Vishnoi G., Kumar R. (2016). Need of standardization of herbal medicines in modern era. Int. J. Phytomed..

[102] Ahmad A., Husain A., Mujeeb M., Siddiqu N., Damanhouri Z., Bhandari A. (2014). Physicochemical and phytochemical standardization with HPTLC fingerprinting of Nigella sativa L. seeds. Pak. J. Pharm. Sci..

[103] Yun Q., Liu Q., He C., Ma X., Gao X., Talbi A., Zhou J. (2014). UPLC-Q-TOF/MS characterization, HPLC fingerprint analysis and species differentiation for quality control of Nigella glandulifera Freyn et Sint seeds and Nigella sativa L. seeds. Anal. Methods.

